# Supplementary material for: The causal relationship between the human gut microbiota and pyogenic arthritis: a Mendelian randomization study
Source: Front Cell Infect Microbiol. 2024 Nov 26;14:1452480. doi: 10.3389/fcimb.2024.1452480 (PMC11629706; doi:10.3389/fcimb.2024.1452480)
Supplement: Supplementary file 1 [file DataSheet1.zip › mendelian test/ebi-a-GCST90027582.csv_forest.pdf]

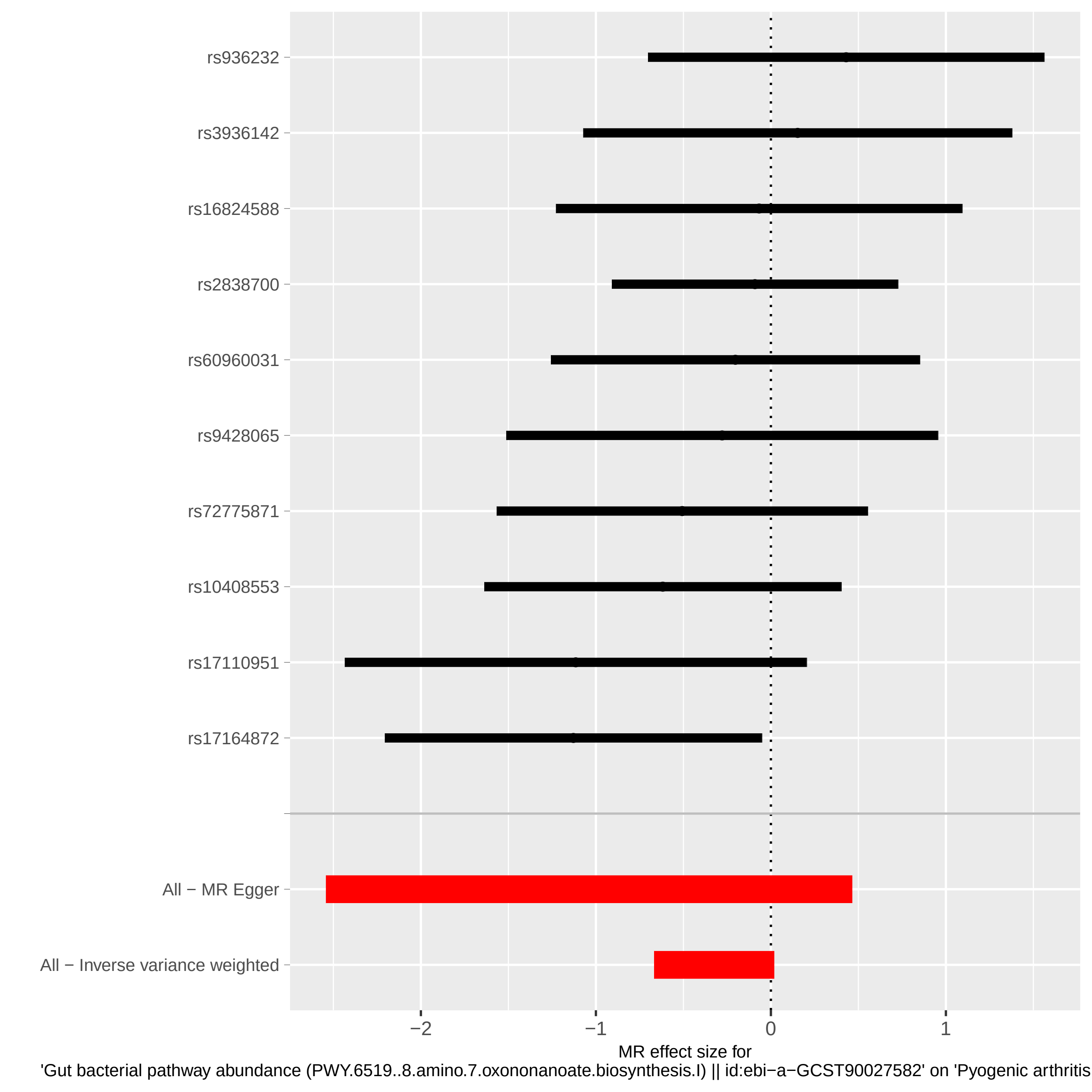

rs936232
rs3936142
rs16824588
rs2838700
rs60960031
rs9428065
rs72775871
rs10408553
rs17110951
rs17164872
All − MR Egger
All − Inverse variance weighted
−2
−1
0
1
MR effect size for
'Gut bacterial pathway abundance (PWY.6519..8.amino.7.oxononanoate.biosynthesis.I) || id:ebi−a−GCST90027582' on 'Pyogenic arthritis
